# Mitochondrial Dysfunction: At the Nexus between Alcohol-Associated Immunometabolic Dysregulation and Tissue Injury

**DOI:** 10.3390/ijms24108650

**Published:** 2023-05-12

**Authors:** Robert W. Siggins, Patrick M. McTernan, Liz Simon, Flavia M. Souza-Smith, Patricia E. Molina

**Affiliations:** 1 Department of Physiology, Louisiana State University Health Sciences Center, New Orleans, LA 70112, USA; rsiggi@lsuhsc.edu (R.W.S.); pmcter@lsuhsc.edu (P.M.M.); lsimo2@lsuhsc.edu (L.S.); fsouz1@lsuhsc.edu (F.M.S.-S.); 2 Comprehensive Alcohol-HIV/AIDS Research Center, Louisiana State University Health Sciences Center, New Orleans, LA 70112, USA

**Keywords:** alcohol, mitochondria, liver, pancreas, adipose tissue, skeletal muscle immunometabolism, innate immunity, adaptive immunity

## Abstract

Alcohol misuse, directly or indirectly as a result of its metabolism, negatively impacts most tissues, including four with critical roles in energy metabolism regulation: the liver, pancreas, adipose, and skeletal muscle. Mitochondria have long been studied for their biosynthetic roles, such as ATP synthesis and initiation of apoptosis. However, current research has provided evidence that mitochondria participate in myriad cellular processes, including immune activation, nutrient sensing in pancreatic β-cells, and skeletal muscle stem and progenitor cell differentiation. The literature indicates that alcohol impairs mitochondrial respiratory capacity, promoting reactive oxygen species (ROS) generation and disrupting mitochondrial dynamics, leading to dysfunctional mitochondria accumulation. As discussed in this review, mitochondrial dyshomeostasis emerges at a nexus between alcohol-disrupted cellular energy metabolism and tissue injury. Here, we highlight this link and focus on alcohol-mediated disruption of immunometabolism, which refers to two distinct, yet interrelated processes. Extrinsic immunometabolism involves processes whereby immune cells and their products influence cellular and/or tissue metabolism. Intrinsic immunometabolism describes immune cell fuel utilization and bioenergetics that affect intracellular processes. Alcohol-induced mitochondrial dysregulation negatively impacts immunometabolism in immune cells, contributing to tissue injury. This review will present the current state of literature, describing alcohol-mediated metabolic and immunometabolic dysregulation from a mitochondrial perspective.

## 1. Introduction

Alcohol misuse has long been recognized to injure nearly every tissue because of its systemic distribution. Metabolic regulatory tissues (liver, pancreas, adipose tissue (AT), and skeletal muscle (SkM)) are particularly impacted by alcohol, with the liver being the most extensively studied [1,2,3,4,5]). However, beyond the well-established liver-centric pathology, alcohol misuse causes pancreatitis, AT inflammation, and loss of SkM mass or sarcopenia. Multiple signaling pathways are disrupted in these tissues, including the dysregulated iron metabolism, which can potentially increase oxidative stress, and this disruption is dependent on the quantity, frequency, and duration of alcohol intake [6,7,8]. Mitochondrial dysfunction has been observed in humans consuming alcohol and in most animal models of alcohol administration [9]. In addition, chronic alcohol misuse leads to persistent systemic inflammation and organ damage [10]. This has led to the overall belief that immune dysregulation is a salient mechanism of alcohol-induced tissue injury. Immune dysregulation is associated with increased production of reactive oxygen species (ROS), leading to oxidative stress. Specifically, alcohol promotes mitochondrial ROS generation, and this has been described as an early process preceding the intrinsic apoptosis pathway [11,12]. Mitochondria produce mostly superoxide anion and hydrogen peroxide, and these molecules play important roles in immune cell function and dysfunction, which has recently been reviewed [13]. Additionally, there is also compelling evidence that increased ROS leads to tissue injury, such as hepatic fibrogenesis [14,15]. However, alcohol-mediated mitochondrial ROS generation and the mechanisms that lead to tissue injury are beyond the scope of this review.

Recently, the intricate link between alcohol-induced dysregulation of immune and metabolic processes has come to the forefront as a potentially important factor contributing to alcohol-associated pathology. This intersection of immunology and metabolism, termed immunometabolism, can be described by two equally important, yet not totally distinct arms of intrinsic and extrinsic immunometabolism [16]. Extrinsic immunometabolism refers to the process whereby immune cells and their products affect cellular, tissue, and whole organism metabolism and involves crosstalk between immune cells and immune signaling from tissue-specific cells. Intrinsic immunometabolism refers to immune cell bioenergetics that influence multiple aspects of immune cell function. Dysregulation of intrinsic immunometabolism can promote pro-inflammatory signaling, affecting metabolic regulatory tissues and, in turn, impacting systemic energy metabolism [17]. We and others propose dysregulated immunometabolism as a central feature of many of the pathological outcomes associated with alcohol misuse (reviewed in [18,19]).

This review discusses the literature on the effects of alcohol on extrinsic immunometabolism in four key metabolic regulatory tissues: liver, pancreas, AT, and SkM. In particular, we discuss the current state of science on mitochondrial-mediated metabolic dysregulation, aberrant cytokine signaling, and tissue–immune cell crosstalk in the context of alcohol use. Additionally, we discuss what is currently known about mitochondria’s role in intrinsic immunometabolism regulating innate (neutrophils and macrophages) and adaptive (T and B) immune cell phenotype and function. Finally, we present evidence of the role of mitochondrial dysfunction as central to alcohol-induced dysregulation of extrinsic and intrinsic immunometabolism and the emergence of a feedforward cycle of ever-increasing tissue damage in the setting of alcohol misuse.

## 2. Impact of Alcohol on Tissues Regulating Metabolic Homeostasis

### 2.1. Liver

Liver is the major site of oxidative metabolism of alcohol, generating the toxic metabolite acetaldehyde, decreasing the NAD+/NADH ratio, and producing excess ROS leading to oxidative stress [20]. Several comprehensive reviews discuss alcohol metabolism and how it leads to alcohol-mediated liver injury [1,18,20,21,22,23]. Central to these processes is mitochondrial function, and alcohol-mediated mitochondrial dysfunction has been studied extensively in alcohol-mediated liver injury, ultimately causing hepatocyte apoptosis, necroptosis, or necrosis [18,24,25,26,27]. 

Alcohol-related liver disease (ARLD) encompasses several conditions with varying degrees of severity and prevalence, including simple steatosis (fatty liver), steatohepatitis, alcohol-related hepatitis, cirrhosis, and increased risk of hepatocellular carcinoma. The most common among these, steatosis, is characterized by hepatocyte lipid droplet accumulation, with no overt inflammation or fibrosis [21]. Among the underlying mechanisms of steatosis is alcohol oxidation. The NADH generated by alcohol metabolism inhibits metabolic pathways, including the Krebs cycle and mitochondrial β-oxidation (reviewed in [21,23,28] Gene transcription of the β oxidation pathway [23] is regulated by peroxisome proliferator-activated receptor alpha (PPARα), a key transcription factor that has been implicated in alcohol-induced injury. In PPARα knock out mice fed 4% alcohol, there was increased hepatic injury including mitochondrial swelling, oxidative stress, inflammation, fibrosis, and apoptosis [29]. Additionally, mice receiving 12 weeks of Lieber-DeCarli liquid diet showed decreased PPARα expression, with hepatic steatosis, necrosis, and inflammatory infiltration indicating a key role of PPARα in alcohol-mediated steatosis and liver injury [30]. A subgroup of these animals receiving the PPARα agonist WY14643 for two weeks showed decreased severity of liver damage, while animals treated with a PPARα antagonist exhibited increased inflammation. PPARα is downstream of 5′-AMP -activated protein kinase (AMPK) and Sirtuin signaling [31]. Alcohol-mediated decreases in PPARα activity may be a result of decreased Sirtuin 1 signaling [32]. Alcohol-mediated decreases in Sirtuin family expression lead to hyperacetylation of several proteins that activate lipogenic pathways and inhibits β-oxidation [33,34,35]. 

Steatosis associated with chronic alcohol misuse can transition to alcohol-related hepatitis, characterized by inflammation and hepatocyte ballooning, with or without fibrosis. In addition to the direct effects of alcohol on the liver, the role of gut-derived pathogen-associated molecular patterns (PAMPs) in the development of alcohol-related hepatitis has been extensively documented [36,37,38]. Gut dysbiosis and leak, together with alcohol-mediated effects on the liver, lead to activation of resident Kupffer cells and other proinflammatory cells to secrete inflammatory cytokines. Thus, immune cells play a significant role in the pathophysiology of alcohol-associated liver injury, and several studies indicate the role of neutrophils, T cells, natural killer cells, and macrophages in the pathogenesis of alcohol-related hepatitis (reviewed in [37,38,39]). Another immune cell type that has recently been shown to contribute to ARLD is mucosa-associated invariant T (MAIT) cells. MAIT cells in the liver, intestinal mucosa, and circulation were significantly decreased in people with severe alcohol-related hepatitis and cirrhosis, and the decrease was associated with decreased antibacterial capacity [40]. 

ARLD exemplifies the cross talk between intra-organ dysregulation and systemic inflammation, thereby bridging metabolic dysregulation to immunometabolic dysfunction. In preclinical models, binge alcohol increased IL-17A production from γδ T cells, and acute-on-chronic alcohol administration increased IL17 production from liver-resident CD4+ T cells. Mechanistically, it was shown that alcohol increased mitochondrial double-stranded RNA in hepatocyte-derived extracellular vesicles that signal to activate Kupffer cells to produce IL-1β, subsequently increasing IL-17 production in γδ T cells [41]. Similarly, in people with ARLD, serum IL-17F was significantly increased and, together with IL-22, was associated with a higher Model For End-Stage Liver Disease (MELD) score [42]. Moreover, Treg cells decreased during alcohol-related hepatitis [43]. Using gene deconvolution with single-cell RNA sequencing data from peripheral immune and dissociated liver cells isolated from severe alcohol-related hepatitis patients, Kim et al. were able to infer a loss of hepatic zonation with fewer central vein hepatocytes and increased inflammatory macrophages [44]. The chronic immune activation and associated chronic inflammation and tissue injury result from the vicious cycle of increased production of alcohol metabolites, oxidative stress, and activation of hepatic stellate cells and cholangiocytes [45,46]. Together, these changes result in dysregulated extracellular matrix deposition and fibrosis, ultimately progressing to cirrhosis in a subset of patients [47,48].

Compelling evidence points to a direct effect of alcohol-mediated mitochondrial dysfunction to promote hepatic injury and disease. In a rat model of chronic alcohol feeding, steatosis, inflammation, and necrosis were all decreased in animals receiving adenoviral vectors containing a mitochondrial-targeted manganese superoxide dismutase (Mn-SOD; [49]). In a similar study of chronic ethanol feeding in the setting of a high-fat diet, nanoparticle delivery of SOD1 was able to prevent alcohol-induced liver damage, partially through downregulation of cytochrome P450 (CYP)2E1 [50]. Alcohol withdrawal in chronic alcohol-fed mice improved metabolic programming by upregulating several key metabolic proteins, including phosphorylated AMPK, PPARα, carnitine palmitoyltransferase-1 (CPT-1), and downregulating fatty acid synthase (FAS) and diacylglycerol acyltransferase-2 (DGAT-2), along with increased activity of superoxide dismutase (SOD) and glutathione peroxidase (GSH-px) [51]. Ethanol withdrawal has also been shown to increase an important antioxidant pathway transcription factor, nuclear respiratory factor 2 (Nrf2), within 1 week of alcohol cessation in a binge-on-chronic mouse model [52]. Moreover, S-Adenosyl-L-Methionine (SAM) prevented an alcohol-mediated decrease in mitochondrial state 3 respiration and cytochrome c oxidase activity, normalized sensitivity for calcium-induced mitochondrial swelling, and prevented the increased sensitivity to nitric oxide (NO)-dependent inhibition of respiration [53]. Studies show that mitochondrial methionine adenosyltransferase a1 (MATa1), an enzyme involved in SAM synthesis, is selectively depleted in ARLD [54]. Additionally in a mouse model fed a Lieber-DeCarli ethanol diet for 4 weeks, GKT137831, a NOX4 inhibitor, increased β-oxidation enzymes, decreased apoptosis, increased mitochondrial membrane potential (ΔΨm), ATP levels and NAD+/NADH ratio, and decreased alcohol-mediated lipid accumulation [55]. In a mouse model of chronic alcohol feeding that decreased hepatic Sirtuin 1 and 3 expression, mitochondrial DNA content, ATP concentrations and hyperacetylated PPARγ coactivator (PGC)-1α, it was shown that dihydromyricetin, a flavonoid, improved hepatic bioenergetics, metabolism, and mitochondrial health [56]. These findings provide strong evidence that alcohol-induced hepatic mitochondrial dysfunction, and the associated dysregulation of immune cell profiles and immunometabolism play important roles in alcohol-mediated liver injury.

### 2.2. Pancreas

Alcohol is a significant risk factor in the development of alcohol-related pancreatitis. The non-oxidative alcohol metabolite fatty acid ethyl esters (FAEEs) are major contributors to alcohol-related pancreatitis and increase mitochondrial dysfunction, decrease ATP production, and impair mitochondrial bioenergetic measures in the pancreas [57,58,59,60,61]. As seen with other metabolically active tissues, mitochondria are critical cell organelles that are affected by alcohol in the pancreas. In rats fed a 14-week Lieber-DeCarli diet, the pancreas was characterized by enlarged and injured mitochondria, decreased apoptotic signaling, pericellular fibrosis, and decreased inflammation, which was attributed to activation of PPARγ [62]. Both ex vivo and in vivo models of pancreatitis showed that oxidative alcohol metabolism activates mitochondrial permeability transition pore (MPTP) opening, leading to decreased ATP production and increased cell death, and that the primary mechanism was decreased NAD+/NADH ratio [63]. In fact, a MPTP inhibitor (TRO40303) was able to protect pancreatic acinar mitochondria and decrease cell death in alcohol-treated pancreatic acinar cells [64]. Chronic alcohol inhibited mitochondrial thiamin pyrophosphate (TPP) uptake by decreasing expression and promoter activity of mitochondrial TPP transporter (SLC25A19) in pancreatic acinar cells, leading to pancreatic injury. Thiamin is necessary for cellular bioenergetic needs [65], particularly for cells of the immune system, and thiamine deficiency results in increased plasma glucose and decreased circulating insulin in the mechanisms of alcohol-induced mitochondrial dysfunction through impaired thiamin regulation [66,67]. In addition to mitochondrial dysfunction, alcohol-related pancreatitis is characterized by infiltration of neutrophils, monocytes, dendritic cells, and T and B lymphocytes [68]. However, the direct role of immune cells in alcohol-related pancreatitis is not mechanistically established, and while there is ample evidence of immunometabolic dysregulation in pancreatic cancer, there is a gap in the literature on the role of impaired immunometabolism as a contributor to alcohol-related endocrine pancreatic dysfunction [69,70,71]. 

### 2.3. Adipose

Adipose tissue (AT) stores excess nutrients in the form of triglycerides (TGs), and through lipolysis, and supplies fuel substrates to other tissues. There are predominantly 3 types of AT, (1) white adipose tissue (WAT) that stores TGs, (2) brown adipose tissue (BAT) that contributes to thermogenesis and maintenance of body temperature [72], and (3) beige adipocytes, located within WAT, which can act as WAT or BAT depending on environmental cues [73]; these include alcohol. Chronic alcohol intake impacts WAT and BAT, leading to lipodystrophy and promoting fat accumulation in peripheral organs, such as the liver (reviewed in [4]). 

In addition to providing cellular energy for the major adipocyte functions of lipid and glucose metabolism, adipocyte mitochondria have multiple critical physiological roles, including adipocyte differentiation, lipid homeostasis, oxidative phosphorylation (OXPHOS), insulin sensitivity, as well as adaptive thermogenesis and WAT browning [74]. White adipocyte mitochondrial dysfunction increases ROS production, decreases ATP production, impairs insulin sensitivity, and leads to whole-body energy dysregulation [75]. Alterations in the adipose tissue immune profile, as well as of mitochondrial function, can impact systemic metabolic homeostasis. WAT OXPHOS damage induced by consumption of a high-fat, high-sugar (HFHS) diet in a mouse model was linked to microbiota-mediated matrix metalloproteinase (Mmp)12+ macrophage inflammation and resulted in impaired systemic glucose metabolism [76]. Mice deficient in adipocyte-specific mitochondrial transcription factor A (Tfam) had decreased activity of proteins in complexes I, III, and IV of the electron transport chain (ETC), resulting in inflammation and adipocyte death in WAT, whitening of BAT, lipodystrophy, insulin resistance, hypertension, and cardiac hypertrophy, reflecting the importance of AT mitochondria in systemic metabolism and the cardiovascular system [77]. 

Macrophages, eosinophils, T cells, and B cells in AT produce cytokines and chemokines that play a significant role in adipocyte mitochondria function, affecting ETC, pyruvate and β-oxidation, branch-chain amino acid metabolism, oxidative stress, and apoptosis [78]. In addition, adipocytes secrete chemokines and cytokines that can initiate and potentiate an influx of activated pro-inflammatory immune cells, further influencing mitochondrial function [79]. In AT, activation of CYP2E1-mediated ethanol metabolism results in increased oxidative stress and impaired adiponectin secretion [80,81]. Furthermore, in a mouse model of chronic ethanol feeding, adipocyte CYP2E1-mediated ethanol metabolism resulted in apoptosis in a BH3 interacting domain death agonist (Bid)-dependent pathway, initiating AT inflammation [82]. Notably, the inflammatory response included a phenotypic shift from anti-inflammatory M2 macrophages towards pro-inflammatory M1 macrophages. Mice administered chronic binge alcohol displayed a trend of increased CD4+ T cells and decreased Tregs in the stromal vascular fraction (SVF), which could potentially increase AT inflammation [83]. In contrast, chronic alcohol feeding increased Tregs in the perilymphatic adipose tissue (PLAT) [84], which may reflect a compensatory response to reduce inflammation-associated tissue injury. Chronic alcohol feeding in rats leads to AT inflammation, decreased adiponectin and visfatin secretion, impaired insulin signaling, and decreased glucose uptake [85]. These alcohol-mediated AT metabolic impairments are adipose depot-specific and more pronounced in visceral AT (VAT) compared to subcutaneous AT (SAT) [85]. Accordingly, AT mitochondrial respiration is unchanged in SAT but decreased in the VAT of obese individuals with fatty liver and NAFLD associated with lower VAT insulin sensitivity [86]. Altogether, these studies suggest an important role of depot-specific AT mitochondrial-mediated energy metabolism and its contribution to insulin resistance and hepatic lipid accumulation, known pathologies attributed to alcohol misuse. The role of mitochondria in adipocyte biology, as impacted by alcohol, warrants further investigation, particularly as it pertains to alterations in extrinsic and intrinsic immunometabolism.

### 2.4. Skeletal Muscle

Skeletal muscle has high bioenergetic requirements and is rich in mitochondria. Alcohol mediates mitochondrial dysfunction and dysregulates functional SkM mass through both direct and indirect effects. Evidence of alcohol-mediated SkM mitochondrial dysfunction is mixed. Earlier studies indicated that alcohol-related dysfunctional SkM mass is not associated with decreased bioenergetic supply and ETC complex activity [87,88]. However, subsequent studies indicate mitochondrial dysfunction with alcohol use. In people with alcohol misuse, cytochrome c oxidase enzyme activity and mitochondrial volume was decreased, and myotubes treated with in vitro ethanol decreased cellular respiration and ETC activity [88,89]. Male and female mice fed a high-fat diet and alcohol exhibited decreased SkM complex I and III activity, with an associated decrease in antioxidant activity [90]. Additionally, chronic alcohol feeding in rats impaired mitochondrial fusion through decreased MFN1 expression, and the sustained loss of mitochondrial fusion impaired bioenergetics and excitation–contraction coupling [91]. In a C. elegans model of alcohol administration, it was shown that alcohol decreased muscle strength, induced expression of antioxidant genes, led to fragmentation of mitochondrial networks, and activated the mitochondrial unfolded protein response [92]. A significant amount of work has been conducted to explore the effect of alcohol in the context of simian immunodeficiency virus (SIV)/HIV on SKM mitochondrial homeostasis. In the preclinical model of SIV infection, chronic binge alcohol increased expression of inflammatory cytokines, decreased antioxidant capacity, and dysregulated several genes implicated in mitochondrial function in antiretroviral therapy (ART)-naïve rhesus macaques [93,94,95]. With effective ART, there is a significant decrease in overt SKM wasting that is characteristic of acquired immunodeficiency syndrome (AIDS). However, alcohol produces cellular and molecular changes that can dysregulate functional SKM mass. Alcohol decreases succinate dehydrogenase activity in type 1 and 3 fibers and myoblast maximal oxygen consumption rate (OCR) [96]. Acute in vitro alcohol decreased glycolytic function, and the alcohol-induced decreased glycolytic function was associated with decreased myoblast differentiation [97]. In people with HIV (PWH) with alcohol use, myoblast respiratory measures that are negative indicators of bioenergetic health, including proton leak and nonmitochondrial respiration, were increased [98]. Thus, there is significant evidence that alcohol affects SkM mitochondrial function. Though alcohol-mediated SkM anabolic and catabolic pathways have been extensively investigated, the role of immune cells and immunometabolism in the context of alcohol-mediated SKM functional mass has not been investigated [4,99,100,101]. Future studies are warranted to identify how extrinsic and intrinsic immunometabolism modulates functional SkM mass, especially in the context of alcohol misuse. 

**Figure 1:**
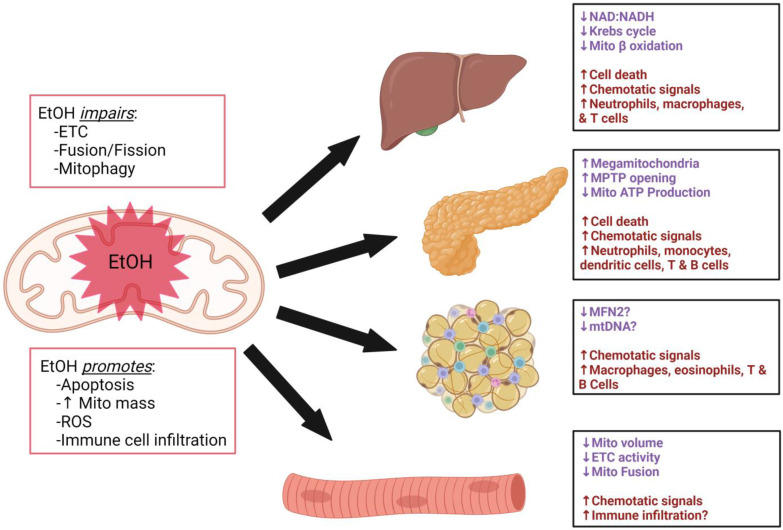
Alcohol-mediated mitochondrial dysregulation in metabolic regulatory tissues. Alcohol (EtOH) impairs electron transport chain (ETC) function, mitochondrial fusion/fission, and mitophagy, while promoting apoptosis, increasing mitochondrial mass, reactive oxygen species and immune cell infiltration in the illustrated tissues. The effects of alcohol on mitochondria are shown in purple and tissue signaling outcomes are shown in maroon in each box corresponding to the individual tissues.

Compelling evidence supports alcohol-mediated dysregulation of extrinsic immunometabolism with mitochondria-centric alterations in the metabolic regulatory tissues (Figure 1). Systematic mechanistic studies are warranted to explore the effect of alcohol in these tissues, particularly in the context of calorie-dense diets. This is relevant because both calorie-dense diets and alcohol independently lead to metabolic dysregulation; however, less is known about the combined effects.

## 3. Impact of Alcohol on Immune Cell Mitochondrial Function

### 3.1. Innate Immune Cells

The innate immune system is the first line of defense against invading pathogens and harmful foreign antigens [102]. A broad interpretation of the innate immune system includes all cells involved in creating the physical barrier between the external and internal environments, bone marrow-derived innate immune cells, and a myriad of secreted chemical mediators. For the purpose of this review, we focus on select innate immune cells—neutrophils and macrophages—due to their known interactions with metabolic regulatory tissues and their well-recognized susceptibility to alcohol-induced dysregulation of maturation, activation, and function [103,104].

#### 3.1.1. Neutrophils

Neutrophils are the most abundant circulating immune cells and represent the first line of defense against foreign antigens. Immature neutrophils rely on mitochondrial OXPHOS for their main source of energy production, and as they mature, these cells undergo extensive mitophagy [105,106,107]. As such, mature neutrophils rely predominantly on glycolysis and have minimal measurable oxygen consumption rate (OCR) [108]. However, mitochondria are instrumental due to their immune functions, including chemotaxis to inflammatory sites, phagocytosis of foreign pathogens, and even the respiratory burst. Neutrophils maintain a measurable ΔΨm through electron transfer from glycolysis to complex III by the glycerol-3-phosphate (G3P) shuttle and mitochondrial glycerol phosphate dehydrogenase (mGPD) [109]. One key function of maintaining ΔΨm is chemotaxis, which is inhibited in experimental systems in which ATP synthase is knocked out or mitochondrial membranes have been chemically uncoupled [110]. During migration, active mitochondria localize to the leading edge of chemotactic neutrophils to provide ATP for local extracellular flux [111,112]. Maintaining ΔΨm is also important for prolonged survival, which is partially mediated through the anti-apoptotic effects of granulocyte colony-stimulating factor (G-CSF) [109,113,114]. Loss of ΔΨm precedes cytochrome c release and activation of the apoptotic program [110,115]. Under pathophysiological conditions, such as infection, glucose supply is often limited and inflamed microenvironments are often hypoxic [116,117], and neutrophils undergo a metabolic shift to glutaminolysis for their energy needs. Glutamine is partially oxidized to glutamate converted to α-ketoglutarate and enters the mitochondrial Krebs cycle, leading to increased NADH levels that contributes to maintaining ΔΨm [117,118]. 

Alcohol has significant effects on neutrophil function. Isolated human peripheral blood and rat peritoneal neutrophils studied one hour after a single alcohol binge, at a dose to achieve 0.1% blood alcohol concentration (BAC), showed increased neutrophil apoptosis that was sustained even 16 h after in vitro ethanol treatment [119]. Chronic 4-month alcohol feeding in rats showed a doubling of neutrophil mitochondrial volume, with increased mitochondrial clumping, swelling, and disrupted cristae [120]. Examination and comparison of peripheral blood cell phenotypes of heavy alcohol drinkers, versus abstinent, or moderate drinkers, and in those with or without infection showed considerable variability in neutrophil frequency, with variations related to infection and not current or previous alcohol use [121]. ROS production was increased, while phagocytosis was impaired after a single binge dose of alcohol in humans [122]. Similarly, in a mouse model of chronic ethanol drinking, neutrophils had decreased migration, activation, and phagocytosis [123].

Extrinsic immunometabolism of neutrophils has been implicated in dysfunction of metabolic regulator tissues in the setting of obesity, diabetes, and alcohol [124,125,126,127,128,129]. Tissue-localized neutrophils undergo metabolic adaptation, with acute glycolytic-mediated ROS production shifting to a more chronic mitochondrial-mediated H2O2 production [124,130]. In a rat model of diabetes, hyperglycemia impaired neutrophil glycolysis accompanied by a compensatory increase in β-oxidation [131]. Mice fed a high-fat diet showed nearly a 20-fold increase in VAT neutrophil accumulation compared to chow-fed controls [132]. Neutrophil accumulation in adipose tissue leads to increased neutrophil elastase production. In non-adipose cell culture models, elastase treatment has been implicated in downregulation of insulin receptor substrate, which could be a contributing factor to insulin resistance observed in metabolic syndrome [133]. Inflamed adipose tissue is not unique in terms of neutrophil infiltration, and patients with acute pancreatitis and ARLD also exhibited increased neutrophil infiltration [134,135]. In people with ARLD, circulating levels of myeloperoxidase (MPO) and neutrophil elastase were significantly increased compared to control subjects. Neutrophil elastase was also positively associated with MELD score [129]. In people with alcohol-related hepatitis, co-localization of neutrophils with apoptotic hepatocytes was prevalent, and there was a strong correlation between the neutrophil infiltration index and hepatocyte apoptosis [136]. Whether this increase in neutrophil infiltration translates to increased tissue levels of neutrophil elastase, and whether insulin signaling mechanisms are impacted in the setting of ARLD, remains unknown.

#### 3.1.2. Macrophages

While neutrophils are the most abundant phagocyte in circulation and migrate to inflamed tissues, macrophages are tissue-resident phagocytes that can markedly impact their tissue microenvironment. Macrophages are metabolically diverse and utilize glycolysis, the Krebs cycle, the pentose phosphate pathway (PPP), β-oxidation, and amino acid metabolism to various degrees depending on differentiation and activation states [137]. Macrophage immunometabolism has been well studied, especially with regard to macrophage polarization [138]. Depending on the inflammatory milieu, naïve macrophages (M0) are induced to undergo polarization to a highly glycolytic pro-inflammatory M1 phenotype or to an OXPHOS-dependent anti-inflammatory M2 phenotype [139]. 

**Figure 2:**
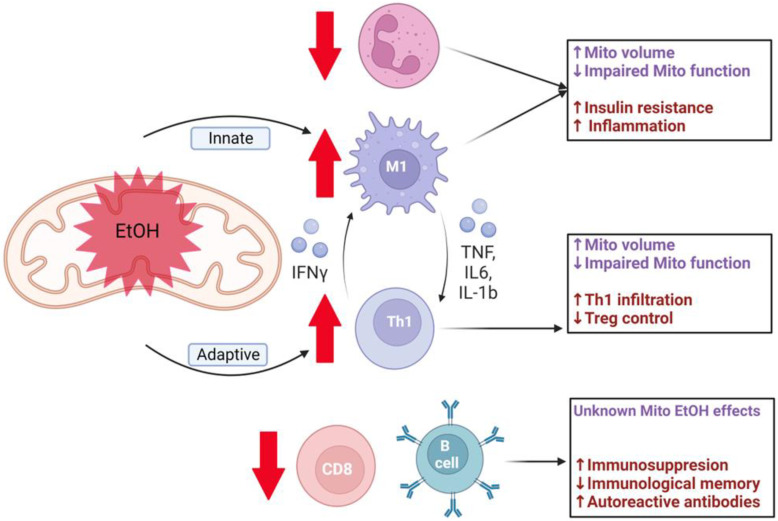
Alcohol-mediated mitochondrial dysregulation contributes to pro-inflammatory environment. Chronic alcohol (EtOH) use impairs mitochondrial function and repair in immune cells, decreases neutrophil, CD8+ T cells, and B cells, and increases pro-inflammatory M1 macrophage and Th1 CD4+ T cells. The effects of alcohol on mitochondria are shown in purple and immune outcomes are shown in maroon in each box corresponding to the individual immune cells. Created with BioRender.com.

During M1 polarization, hypoxia-inducible factor 1-alpha (HIF-1α) is upregulated by Toll-like receptor-4 (TLR-4) signaling, and HIF-1α enhances glycolytic flux potentially through increased GLUT1 and hexokinase 2 transcription [140,141,142,143,144]. Many of the signature pro-inflammatory functions of M1 macrophages are ameliorated in myeloid-specific GLUT1 knockout mice [145]. Activated M1 macrophages produce many pro-inflammatory cytokines, including tumor necrosis factor-alpha (TNFα), interleukin 6 (IL6), and IL1β [146]. These cytokines promote CD4+ T lymphocytes towards a T helper (Th)1 response (discussed below), leading to production of interferon gamma (IFNγ). IFNγ promotes additional M1 polarization, creating a positive feedback loop for as long as the initiating stimulus remains (Figure 2) [138]. We and others have shown that alcohol promotes a Th1 phenotype, and whether ethanol independently and directly promotes an M1 phenotype remains to be determined [147,148]. Even though M1 macrophages are reliant on glycolysis for polarization and function, lipopolysaccharide (LPS) signaling was shown to increase M1 macrophage ΔΨm, which is protective against apoptosis [149]. M2 macrophages are critical to the resolution of inflammatory responses and tissue repair [141] and M2 polarization requires OXPHOS and β-oxidation [138]. IL-4 and/or IL-13 mediate M2 polarization, and this is largely achieved through PGC-1β induction of metabolic programs for β-oxidation and mitochondrial biogenesis [138]. Transgenic expression of PGC-1β in naïve M0 macrophages prevents M1 polarization, highlighting the essential role of immunometabolism in regulating macrophage polarization [150]. 

Similar to neutrophils, alcohol dysregulates macrophage function and polarization in a dose- and frequency-dependent manner. In vitro studies of short- and long-term ethanol treatment of human monocytes showed that acute ethanol exposure (24–48 h) inhibited the inflammatory response to LPS, indicative of an M2 phenotype, and prolonged exposure (7 days) promoted a robust M1 response [151]. Healthy volunteers receiving a single alcoholic drink, to achieve a maximum BAC of <0.04% after 4 h, showed an increase in M2-like circulating monocyte polarization, similar to the observed shift following short-term in vitro alcohol treatment [152]. Alcohol misuse also affects macrophage mitochondrial function in various tissues. In both mouse and rat models of chronic alcohol feeding, liver-resident macrophages were polarized to a pro-inflammatory M1 phenotype [153,154]. Mice fed an alcohol diet for 6 weeks followed by LPS injection showed enhanced M1 macrophage-mediated liver inflammation, and this was further exacerbated in myeloid-specific autophagy-related 7 (Atg7) knockout mice [155]. Interestingly, the macrophages in these autophagy-deficient mice showed an accumulation of defective mitochondria, suggesting M2 polarization could be impaired. 

Intracellular iron concentrations are important to macrophage polarization processes. It has been shown that low iron increases pro-inflammatory M1, likely through Krebs cycle inhibition, and high iron increases anti-inflammatory M2 macrophages [156]. However, the link between iron and macrophage polarization with alcohol use is less clear. Alcohol increases hepatic and circulating iron levels in ARLD [157] Kupffer cells increase their iron uptake, but rather than transitioning to an M2 phenotype, they seem to remain pro-inflammatory. This indicates that iron status and alcohol are potentially having independent effects on mitochondrial bioenergetics and macrophage polarization.

In addition to the liver, the effects of ethanol on alveolar macrophages have also been intensively investigated. In a chronic ethanol-fed mouse model, administration of the PPARγ ligand, rosiglitazone decreased the ethanol-induced expression of NADPH oxidase (NOX) 4 [158]. NOX4 is central to M1 macrophage ROS generation, and PPARγ signaling promotes β-oxidation and OXPHOS (M2 polarization) [150,159]. In nearly all models studied, chronic ethanol appears to promote a tissue pro-inflammatory environment through metabolic programming towards an M1 phenotype, impairing mitochondrial function and thus inhibiting M2 polarization. This nexus between deranged immune cellular energy metabolism and tissue dysfunction emerges as a salient mechanism across multiple organ systems.

### 3.2. Adaptive Immune Cells

The adaptive immune system, consisting of T and B cells, is a critical component of the immune response to remove foreign pathogens and develop immunological memory. T cells are classified as CD4+ and CD8+, based on the expression of an accessory glycoprotein co-receptor, which interacts with the major histocompatibility complex (MHC) Class II and I molecules, respectively, on antigen-presenting cells (APC) [160,161]. B cells differentiate into plasma cells to produce antibodies against a foreign pathogen. CD8+ T cells are responsible for attacking infected and malignant cells. CD4+ T cells, known as T helper (Th) cells, have multiple functions, including recruitment of cytotoxic T cells to sites of infection, coordinating the humoral antibody response (B cells), and regulating both innate and adaptive immune cell activation and proliferation. T and B cells are activated by APCs, and upon activation, immune cell bioenergetics undergoes metabolic transition from a quiescent to highly metabolic state. This is required for the energetic demands needed for clonal expansion in response to infection [162,163]. Mitochondrial function is critical for both activation and cytokine production of CD8+ T cells, while functional mitochondria is required to maintain persistent proliferation after activation of CD4+ T cells and B cells [163,164,165]. After clearance of infection, a small percentage of CD4+, CD8+ T cells and B cells will become memory cells, with a transition back to a quiescent mitochondrial-dependent state, establishing immunological memory to the previously terminated foreign pathogen [165,166].

#### 3.2.1. CD4+ T Cells

CD4+ T cells rely on both glycolysis and OXPHOS to support the necessary energy requirements needed during an immune response [162]. After activation, CD4+ T cells begin a differentiation process into different subsets, as directed by a transcription factor network dependent on distinct metabolic pathways [164,167,168]). Pro-inflammatory Th1, Th2, and Th17 subsets rely on glycolysis, while immunosuppressive T regulatory cells (Tregs) rely on OXPHOS for differentiation. Our group has demonstrated that ethanol dysregulates normal CD4+ T cell differentiation by promoting Th1 from naïve CD4+ T cells [148]. This increase in Th1 CD4+ T cells was associated with an increase in GLUT1 expression, the primary glucose transporter in CD4+ T cells, glucose analog 2-Deoxy-2-[(7-nitro-2,1,3-benzoxadiazol-4-yl) amino]–D-glucose (2-NBDG) uptake, and glycolysis. Further, it was shown that inhibiting glycolysis with 2-deoxyglucose (2DG), prevented an ethanol-mediated increase in Th1 cells. This study also demonstrated that ethanol impaired mitochondrial function and Treg differentiation. Ethanol-treated CD4+ T cells had decreased maximal respiration, coupling efficiency, OCR-linked ATP production, increased proton leak and a decreased bioenergetic health index. These ethanol-mediated impairments to CD4+ T cell mitochondrial function occurred only in differentiating Tregs and were associated with higher mitochondrial content. Ethanol-treated CD4+ T cells had decreased expression of ATG7 gene, a protein important in autophagosome formation during autophagy. We propose that as a result of these ethanol-mediated changes in mitochondrial dynamics, Treg function may be impaired and unable to control the proliferation of pro-inflammatory Th1 cells [148]. Further, in vivo studies in mice have observed similar ethanol-mediated changes to CD4+ T cell subsets with increased Th1:Treg ratio in their colon [147]. These studies highlight the need for future studies to investigate this ethanol-mediated immunometabolic dysregulation, as it may play a significant role in the impaired immune response that characterizes subjects with alcohol misuse. 

#### 3.2.2. CD8+ T Cells

CD8+ T cells also undergo a dynamic shift in cell metabolism and switch from OXPHOS to glycolysis upon activation [167,169]. This transition is necessary to support the growth and differentiation of cytotoxic T cells during infection. Following pathogen clearance, a small percentage of CD8+ T cells will undergo a metabolic shift back to OXPHOS and differentiate into long-lived quiescent memory CD8+ T cells, while the rest undergo cell death. Chronic ethanol exposure in mice leads to decreased CD8+ memory cell response to secondary influenza A virus infection [170]. Further, chronic binge alcohol administration for 3 months in rhesus macaques significantly decreased the percent effector memory CD8+ T cells and resulted in higher percentages of memory CD4+ T cells in the jejunum [171], highlighting potential differential effects of ethanol of CD4+ and CD8+ T cells. Memory CD8+ T cells rely heavily on lipid oxidation [169] and ethanol impairs lipid oxidation in hepatocytes [31]. This ethanol effect on lipid oxidation has yet to be investigated in CD8+ T cells. 

#### 3.2.3. B Cells

Like T cells, non-activated B cells exist in a quiescent state, and activation initiates metabolic reprogramming to drive re-entry into the cell cycle. Activated B cells upregulate glucose import and increase OCR, but unlike T cells, metabolites generated from glycolysis are shuttled into the PPP [172,173]. Additionally, unlike T cells, glucose restriction has a minor impact on B cell activation [163]. Taken together, these studies show that activated B cell metabolic reprogramming differs from T cells. Less is known regarding the metabolism of memory B cells. Autophagy is vital to memory B cell maintenance and persistence, as memory B cells were not able to persist in mice deficient in ATG7 [174,175,176]. People with alcohol misuse have lower B cells numbers compared to moderate/light drinkers and individuals with severe ARLD [177,178,179,180]. Knowledge on the impact of ethanol on B cell bioenergetics is also lacking. If ethanol impairs mitochondrial function, as well as ATG7 expression, as observed in CD4+ T cells, it is possible that this decrease in B cells, as well as CD8+ T cells, could be due to disruption of mitochondrial homeostasis caused by ethanol-related mechanisms. In addition to ethanol-mediated damage to hematopoietic tissues, mitochondrial dyshomeostasis may contribute to the decreased circulating immune cell numbers and function that is observed in chronic alcohol misusers [181,182,183].

These alcohol-mediated changes to the CD4+ T cell subsets mimic those we have seen during in vitro ethanol treatment [148] and suggest they are possibly caused by impairments in mitochondrial bioenergetics in T cells. Such impairments to mitochondrial function in Tregs would result in a pro-inflammatory environment in metabolic regulatory tissues, as discussed earlier. These ethanol-mediated changes to intrinsic immunometabolism (Figure 2) within tissues warrant further investigation to help identify therapeutic targets to alleviate the increase in peripheral tissue immune cell infiltration and tissue damage observed in individuals that misuse alcohol.

## 4. Discussion

Alcohol is the most widely misused substance, and alcohol and its metabolites have been implicated in the etiologies of multiple pathophysiological conditions [1]. Every organ system and cell of the body is a potential target of the deleterious effects of alcohol and its metabolites. Metabolism is perhaps the most fundamental process of living organisms, and whole organism metabolism intricately connects all the cells of the body, directly or indirectly, through neuroendocrine and immune signaling. Mitochondria are found in nearly every cell of the body and play a predominant role in metabolic signaling [184]. Numerous lines of evidence support mitochondrial dysfunction preceding many non-communicable diseases, which has been reviewed recently [185]. While progressive mitochondrial damage may lead to further tissue functional impairment and long-term disease progression, there is evidence that interventions such as caloric restriction and exercise enhance mitochondrial function and ameliorate some of the deficits of chronic diseases, such as chronic inflammation obesity, and type 2 diabetes mellitus (T2DM) [186,187,188]. Clinical and preclinical studies show that alcohol impairs mitochondrial function [9]. There is compelling evidence that chronic alcohol consumption mediates mitochondrial damage, leading to the progressive development of T2DM [189]. A strong link between alcohol-induced mitochondrial damage underpinning disease pathogenesis was elucidated recently in a study showing that hepatocytes from ARLD patients are selectively depleted in MATα1, and preserving mitochondrial MATα1 content prevented alcohol-induced mitochondrial dysfunction [54].

Alcohol-mediated impairments to metabolic regulatory tissues, such as those covered in the current review, lead to both altered microenvironments and to whole organism metabolic derangement. Many of the deleterious effects of alcohol on whole organism metabolism can be linked to damaged tissue mitochondria. Importantly, tissue mitochondrial damage leading to cell death releases systemic signals to promote chemotaxis of immune cells to those sites of injury [190]. This sets the stage for pro-inflammatory immune cell infiltration within the injured tissues. Thus, with alcohol, the infiltrating immune cells are primed for pro-inflammatory phenotypes both through deranged systemic metabolic signals, e.g., hyperglycemia, and directly via alcohol-mediated immunometabolic disruption of immunometabolism. 

**Figure 3:**
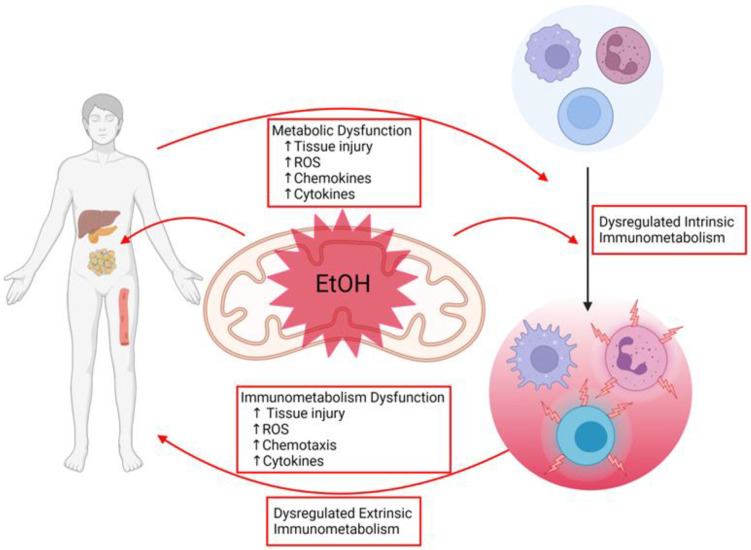
Alcohol-mediated mitochondrial dysfunction is at the nexus of extrinsic and intrinsic immunometabolic adaptations. Ethanol-mediated impairment of mitochondrial function increases metabolic dysfunction in tissue, which dysregulates intrinsic immunometabolism, leading to immunometabolism dysfunction and thus dysregulated extrinsic immunometabolism with alcohol misuse. Created with BioRender.com.

As has been discussed, alcohol disrupts intrinsic immunometabolism, largely through impairment of mitochondrial signaling and OXPHOS, promoting pro-inflammatory phenotypes in both innate and adaptive immune cells. These alcohol-primed pro-inflammatory immune cells undergo chemotaxis to metabolic regulatory tissues that are also susceptible to alcohol-induced injury. The extrinsic immunometabolic effects of these immune cells and their inflammatory mediators will further impair or dysregulate host tissue mitochondria, contributing to exacerbated whole-organism metabolic dyshomeostasis. This alcohol-mediated mitochondrial dysfunction is central to creating a vicious cycle of pro-inflammatory immune cell infiltration and tissue destruction caused by immune cells (Figure 3). Elucidating how alcohol causes these changes to immune cell intrinsic immunometabolism and, as a result, produces deleterious effects on extrinsic immunometabolism will elucidate key mechanisms that can be targeted to alleviate the vicious cycle of alcohol-mediated end organ injury and metabolic dysfunction.

## 5. Conclusions

Alcohol misuse causes deleterious effects in every tissue. However, there are no single unifying mechanisms for alcohol’s pathological effects. Because mitochondrial function under healthy physiological conditions is tailored to tissue metabolic needs, it stands to reason that alcohol-impaired mitochondrial function is a critical contributor to tissue injury. As discussed in this review, mitochondrial damage is a key effector of metabolic tissue dysfunction, and mitochondrial dysfunction underlies considerable dysregulation of immune function through aberrant intrinsic and extrinsic immunometabolism. The pervasive damaging effects of alcohol, in part, can thus be attributed to the detrimental crosstalk between impaired metabolic tissue signaling and immune cell function, with mitochondrial dysfunction at the nexus of this interaction.

## Data Availability

Not applicable.

## Abbreviations

Abbreviations used in this paper: 2-NBDG—2-Deoxy-2-[(7-nitro-2,1,3-benzoxadiazol-4-yl) amino]-D-glucose; 2-DG—2-Deoxyglucose; AMPK—5’ adenosine monophosphate-activated protein kinase; AIDS—acquired immunodeficiency syndrome; ATP—Adenosine triphosphate; AT—Adipose Tissue; ARLD—Alcohol-related liver disease; APC—antigen-presenting cells; ART—antiretroviral therapy; ATG7—autophagy-related 7; Bid—BH3 interacting domain death agonist; BAC—blood alcohol concentration; BAT—brown adipose tissue; CPT-1—carnitine palmitoyltransferase-1; CYP—cytochrome P450; DNA—deoxyribonucleic acid; DGAT-2—diacylglycerol acyltransferase-2; ETC—electron transport chain; FAEEs—fatty acid ethyl esters; FAS—fatty acid synthase; GLUT1—glucose transporter 1; GSH-px—glutathione peroxidase; G3P—glycerol-3-phosphate; G-CSF—granulocyte colony-stimulating factor; HFHS—high-fat, high-sugar diet; HIV—Human immunodeficiency virus; H2O2—hydrogen peroxide; HIF-1α—hypoxia-inducible factor 1-alpha; IFNγ—interferon gamma; IL—interleukin; LPS—lipopolysaccharide; Mn-SOD - manganese superoxide dismutase; MHC—major histocompatibility complex; Mmp—matrix metalloproteinase; mGPD—mitochondrial glycerol phosphate dehydrogenase; MATa1—mitochondrial methionine adenosyltransferase a1; MPTP—mitochondrial permeability transition pore; Tfam—mitochondrial transcription factor A; MFN1—Mitofusin 1; MELD—Model for End-Stage Liver disease; MAIT—mucosa-associated invariant T cells; MPO—myeloperoxidase; NOX4—NADPH Oxidase 4; NAD(H)—Nicotinamide Adenine Dinucleotide; NADP(H)—Nicotinamide adenine dinucleotide phosphate; NO—nitric oxide; Nrf2—nuclear respiratory factor 2; OXPHOS—oxidative phosphorylation; OCR—oxygen consumption rate; PAMPs—pathogen-associated molecular patterns; PPP—pentose phosphate pathway; PWH—people with HIV; PLAT—perilymphatic adipose tissue; PPARα—peroxisome proliferator-activated receptor alpha; PGC-1α—PPARγ coactivator-1α; PGC-1β—PPARγ coactivator-1β; ROS—reactive oxygen species; Tregs—regulatory T cells; SAM—S-Adenosyl-L-Methionine; SIV—simian immunodeficiency virus; SkM—skeletal muscle; SVF—stromal vascular fraction; SAT—subcutaneous adipose tissue; SOD—superoxide dismutase; Th—T helper; TPP. thiamin pyrophosphate; TLR-4—Toll-like receptor-4; TGs—triglycerides; TNFα—tumor necrosis factor-alpha; T2DM—type 2 diabetes mellitus; VAT—visceral adipose tissue; WAT—white adipose tissue.
